# Functional Characterization of *Plasmodium falciparum* Surface-Related Antigen as a Potential Blood-Stage Vaccine Target

**DOI:** 10.1093/infdis/jiy222

**Published:** 2018-04-18

**Authors:** Emmanuel Amlabu, Henrietta Mensah-Brown, Prince B Nyarko, Ojo-ajogu Akuh, Grace Opoku, Philip Ilani, Richard Oyagbenro, Kwame Asiedu, Yaw Aniweh, Gordon A Awandare

**Affiliations:** 1 West African Center for Cell Biology of Infectious Pathogens, Department of Biochemistry, Cell and Molecular Biology, College of Basic and Applied Sciences, University of Ghana, Legon, Accra; 2 Department of Biochemistry, Kogi State University, Anyigba, Nigeria

**Keywords:** *Plasmodium falciparum*, malaria vaccine, erythrocyte invasion, novel antigens, naturally acquired immunity

## Abstract

*Plasmodium falciparum* erythrocyte invasion is a multistep process that involves a spectrum of interactions that are not well characterized. We have characterized a 113-kDa immunogenic protein, PF3D7_1431400 (PF14_0293), that possesses coiled-coil structures. The protein is localized on the surfaces of both merozoites and gametocytes, hence the name *Plasmodium falciparum* surface-related antigen (*Pf*SRA). The processed 32-kDa fragment of *Pf*SRA binds normal human erythrocytes with different sensitivities to enzyme treatments. Temporal imaging from initial attachment to internalization of viable merozoites revealed that a fragment of *Pf*SRA, along with *Pf*MSP1_19,_ is internalized after invasion. Moreover, parasite growth inhibition assays showed that *Pf*SRA P1 antibodies potently inhibited erythrocyte invasion of both sialic acid–dependent and –independent parasite strains. Also, immunoepidemiological studies show that malaria-infected populations have naturally acquired antibodies against *Pf*SRA. Overall, the results demonstrate that *Pf*SRA has the structural and functional characteristics of a very promising target for vaccine development.

## Teaser

We have identified and functionally characterized a novel *Plasmodium falciparum* surface-related antigen (*Pf*SRA) as a potential multistage vaccine candidate. The antigen is localized on both merozoites and gametocytes with high anti-*Pf*SRA growth inhibition assay activity in laboratory strains and clinical isolates.

Malaria is a deadly infectious disease that affects inhabitants of the tropics and subtropical regions of the world and accounts for approximately 212 million cases and 429 000 deaths annually [1]. The clinical manifestation of the disease begins from the blood stage of the infection, during which the parasite invades human erythrocytes [2]. *Plasmodium falciparum* erythrocyte invasion is a complicated process that involves an array of receptor–ligand interactions [3] and/or protein–protein interactions [4–8] that occur at the parasite–host cell interface and facilitate the recruitment of the parasite’s invasion machinery.

Thus, recent identification of *P. falciparum* surface proteins that are accessible to both humoral and cellular immune systems is a major advancement toward vaccine development against malaria [9–12]. This has given great impetus to the idea of a multiantigen vaccine as an intervention strategy against blood-stage malaria. Therefore, the selection and prioritization of candidate antigens are critical aspects of this strategy. Although several blood-stage antigens have been extensively studied, few have demonstrated the desired qualities for a vaccine candidate. One of the remarkable observations from the *P. falciparum* genome sequencing project was that nearly 60% of the parasite’s genes lacked sequence similarity to genes from other known organisms, and thus these genes have remained hypothetical with no defined functional roles [13]. Subsequently, the availability of more comprehensive genomic, proteomic, and transcriptomic datasets from both humans and *Plasmodium* has paved the way for further characterization of these hypothetical proteins using informatics-based approaches. This is required for successful identification of a repertoire of novel *P. falciparum* merozoite antigens that could be explored as targets for a rational vaccine design [14].

A detailed understanding of the functional roles of *P. falciparum* novel merozoite antigens, their localization, and their fate during invasion is critical to the identification of targets of host immunity and prioritization of merozoite antigens for inclusion in blood-stage malaria vaccines.

Herein, we have identified a novel *P. falciparum* protein (PlasmoDB ID: PF3D7_1431400/PF14_0293) that we have named *P. falciparum* surface-related antigen (*Pf*SRA), based on its dual subcellular localization on both merozoites and gametocytes. Native *Pf*SRA is proteolytically processed into multiple fragments in parasite culture supernatant, and the 32-kDa fragment of *Pf*SRA exhibits erythrocyte-binding activity. More important, antibodies against *Pf*SRA potently inhibited merozoite invasion of erythrocytes by both sialic acid–dependent and sialic acid–independent parasite strains. The data also demonstrated that *Pf*SRA is a target for naturally acquired immune responses in humans.

## METHODS

### Screening for New Blood-Stage Vaccine Candidates

To identify new blood-stage proteins as potential vaccine candidates, a systematic screening procedure was implemented. This included analysis of temporal gene expression relative to other well-characterized invasion-type genes and in silico interrogation of protein structural features. Once all of these selection criteria were ascertained, sequence alignment analysis was done to evaluate the conservation level of the target gene across the different *Plasmodium* species orthologs. Finally, we scanned the entire protein sequence using a current state-of-the-art online threading program to identify coiled-coil regions [15].

### Peptide Synthesis and Immunogenicity Studies

Three synthetic peptides corresponding to the immunogenic epitopes were synthesized by GeneScript on the basis that they harbor coiled-coil signatures corresponding to the conserved regions in *Pf*SRA orthologs.

### Plasma Samples and Immunoreactivity

Ethical approval was obtained from the ethics committees as documented previously [16, 17]. Immunoreactivity screenings of plasma samples were performed by enzyme-linked immunosorbent assay (ELISA) [12] and immuno-dot blotting [18] using the synthetic peptides *Pf*SRA P1 (NNKDNHNKKDTNENC); *Pf*SRA P2 (CENDNDEYGNKNKNS); and *Pf*SRA P3 (CSNNKKKKK NDKKKK). R1 peptide (LFSKFGSRMHILKC) was used as control (R1 peptide blocks AMA 1–RON 4 interaction), and naive plasma was used as negative control.

### Affinity Purification of *Plasmodium falciparum* Surface-Related Antigen C-Terminal Human Antibodies

The C-terminal α-*Pf*SRA human antibodies (α-*Pf*SRA P3) were affinity-purified from plasma samples of malaria-exposed children as described previously [19].

### Parasite Culture Supernatant, Ring-Stage Invasion Supernatant, and Erythrocyte-Binding Assay

*Plasmodium falciparum* strains 3D7 and W2mef were maintained in culture as described previously [16]. Schizonts were purified using Percoll-alanine gradient centrifugation [20], followed by saponin lysis, and the recovered parasite pellets were further lysed in sodium dodecyl sulfate-polyacrylamide gel electrophoresis sample buffer. Parasite culture supernatant or ring-stage invasion supernatant were used as the source for native *Pf*SRA, which was detected by immunoblotting using primary α-rabbit antibodies to the 3 peptides (α-*Pf*SRA P1, α-*Pf*SRA P2, and α-*Pf*SRA P3). The immunoblots were developed using goat α-rabbit horseradish peroxidase–conjugated, secondary antibody, and enhanced chemiluminescence reagents (Thermo Scientific). Erythrocyte-binding assay (EBA) was performed using parasite culture supernatant as described previously [21].

### Permeabilized and Nonpermeabilized Immunofluorescence Assays

Immunofluorescence microscopy was carried out using *P. falciparum* (3D7)–infected erythrocytes smeared onto glass slides and fixed in prechilled methanol for 30 minutes. Fixed erythrocytes were permeabilized using 0.1 % Triton X-100 formulated in phosphate-buffered saline. Nonpermeabilized, liquid immunofluorescence assay (IFA) was carried out as described previously [22]. After the washing step for both IFA conditions, the slides were blocked for 1 hour in PBS containing 3% bovine serum albumin. Slides were probed with primary and secondary antibodies for the respective antigens and mounted in vectashield (Vector Laboratories Inc) supplemented with 4′,6-diamidino-2-phenylindole for staining the nucleus. Fluorescence microscopy was performed on an Olympus fluorescence microscope (BX41). Images captured were processed using the open access Fiji-Image J software (National Institutes of Health).

### Antibody Internalization Assay

To test for the internalization of α-*Pf*SRA P3 antibodies, α-*Pf*SRA P3 and α-*Pf*MSP-1_19_ polyclonal antibodies were incubated with tightly synchronized segmenting schizonts that were allowed to rupture and release merozoites. The viability of the released merozoites was assessed at the different stages (early, mid, and late) of an invading merozoite. For each stage, smears were prepared, fixed, and examined by immunofluorescence microscopy as described above.

### Growth Inhibition Assays

Growth inhibition assays (GIAs) were performed as described previously [23], and parasitemia levels were determined using flow cytometry on a BD FORTESSA X-20 with Flo J software. Erythrocyte invasion inhibitory effects of the α-*Pf*SRA antibodies were estimated by comparison of percentage of invasion of controls with test assay.

## RESULTS

### Identification of *Plasmodium falciparum* Surface-Related Antigen as a Potential Malaria Vaccine Candidate

In a systematic screen of uncharacterized *P. falciparum* proteins for potential blood-stage vaccine candidates, we performed data-mining analysis for genes with peak mRNA expression levels in late schizogony using data from *P. falciparum* transcriptome studies [24, 25] and another study on the prediction of PfSUB-1 protease specificity [26]. The details of all analyses have been described in the Supplementary Material (Supplementary Figure 1*A* and 1*B*). Overall, *Pf*SRA emerged as the top hit with both signal peptide and a predicted glycosylphosphatidylinositol attachment site.

Furthermore, we generated sequence alignments with other orthologs of *Pf*SRA and showed that the C-terminus of *Pf*SRA had 5 positionally conserved cysteine residues across the different *Plasmodium* species orthologs (Supplementary Figure 2). Additional predictive analysis from PSI-Pred revealed that the *Pf*SRA protein sequence harbored coiled-coil signatures (Supplementary Figure 3). This signature forms stable structures that elicit functional antibodies and was considered as a basis for the design of 3 *Pf*SRA chemically synthesized peptides used for antibody generation.

### Induction of *Plasmodium falciparum* Surface Related Antigen-Specific Antibodies Using Synthetic Peptides

**Figure 1.**
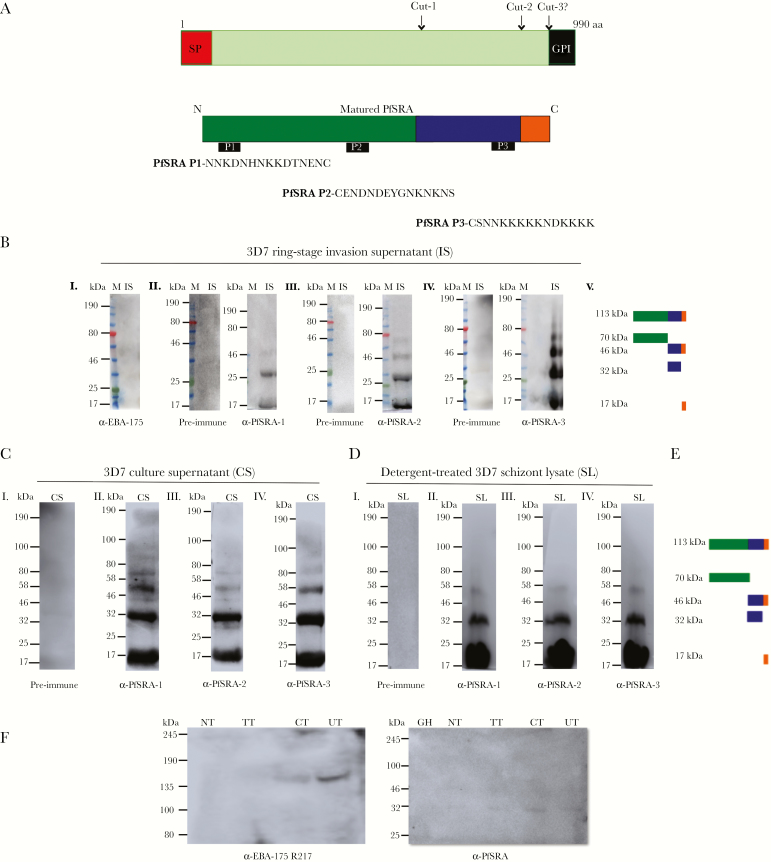
Domain organization and proteolytic processing of native *Plasmodium falciparum* surface-related antigen (*Pf*SRA)*. A*, *Pf*SRA possesses a signal peptide and a predicted glycosylphosphatidylinositol (GPI) anchor. Cut-1 and -2 represents *Pf*SUB-1 cleavage sites analyzed by the Prediction of Protease Specificity tool. Cut-3 represents the GPI–transamidase cleavage site for the predicted GPI attachment signal. *Pf*SRA P1, *Pf*SRA P2, and *Pf*SRA P3 designate conserved regions of *Pf*SRA in different orthologs that possesses coiled-coil signatures for which chemically synthesized peptides were designed. C designates the carboxyl terminus, and N designates the amino terminus. *B*, α-*Pf*SRA antibodies detect multiple processed fragments of native *Pf*SRA during immunoblotting of 3D7 ring-stage invasion supernatants (ISs) that were probed with mouse α-EBA-175 (R217) antibody (I), rabbit α-*Pf*SRA P1 antibody (lot no.: A417040387) (II), α-PfSRA P2 antibody (lot no.: A417040332) (III), and α-*Pf*SRA P3 antibody (lot no.: A417040334) (IV). The colored bars at the far right represent the predicted processed fragments (70, 58, 32, and 17 kDa) (V). *C*, Anti-*Pf*SRA antibodies detect multiple processed fragments of native *Pf*SRA during immunoblotting of 3D7 parasite culture supernatants (CSs) that were probed with preimmune sera (I), α-*Pf*SRA P1 antibody (II), α-*Pf*SRA P2 antibody (III), and *Pf*SRA P3 antibody (IV). *D*, Anti-*Pf*SRA antibodies detect multiple processed fragments of native *Pf*SRA during immunoblotting of 3D7 schizont lysates (SLs) that were probed with preimmune sera (I), α-*Pf*SRA-P1 antibody (II), α-*Pf*SRA-P2 antibody (III), and *Pf*SRA-P3 antibody (IV). *E*, The colored bars at the far right represent the predicted processed fragments (70, 58, 32, and 17 kDa) (V). *F*, Recombinant EBA-175 was used as a control, and it bound erythrocytes in a neuraminidase-sensitive, trypsin-sensitive, and chymotrypsin-resistant manner. Similarly, the 32-kDa processed fragment of native *Pf*SRA bound erythrocytes in a neuraminidase-sensitive, trypsin-sensitive, and chymotrypsin-resistant manner. Abbreviations: CT, chymotrypsin; GH, normal human erythrocyte ghost; NT, neuraminidase; TT, trypsin; UT, untreated control.

Despite several optimization procedures for expression in *Escherichia coli*, attempts to express recombinant *Pf*SRA protein were unsuccessful. However, we designed 3 peptides for synthesis that corresponded to the conserved regions of *Pf*SRA in other *Plasmodium* species orthologs (Figure 1A). These *Pf*SRA-derived peptides (*Pf*SRA P1, *Pf*SRA P2, and *Pf*SRA P3) include coiled-coil signatures (Supplementary Figure 3). Immunization of rabbits with the 3 synthetic peptides (*Pf*SRA P1, *Pf*SRA P2, and *Pf*SRA P3) by Genescript resulted in high titers of *Pf*SRA peptide-specific antibodies, which were used in subsequent experiments.

### Proteolytic Processing and Erythrocyte Binding Activity of *Plasmodium falciparum* Surface-Related Antigen

Preimmune sera were used for all immunoblotting as a negative control that did not detect native *Pf*SRA. As expected, α-EBA-175 (R217) antibodies did not detect native EBA-175 under reduced condition (Figure 1B, I). The antibodies (α-*Pf*SRA P1, α-*Pf*SRA P2, and α-*Pf*SRA P3) consistently detected multiple processed fragments (17, 32, and 58 kDa) of the native *Pf*SRA in ring-stage invasion supernatants (Figure 1B, II–V) or parasite culture supernatants (Figure 1C, I–IV) and schizont lysates (Figure 1D, I–IV).

To identify the fragment(s) of *Pf*SRA that possess erythrocyte-binding activity, we performed erythrocyte binding assays. The 32-kDa fragment of *Pf*SRA was clearly detected in the eluate, suggesting that it bound erythrocytes. Under higher exposure, the 113-kDa full-length *Pf*SRA and the 58-kDa processed fragment were also detectable (Supplementary Figure 3), suggesting a weaker binding relative to the 32-kDa fragment. Of interest, the binding was sensitive to neuraminidase and trypsin treatments but resistant to chymotrypsin treatment (Figure 1F). As a control, recombinant EBA-175 (R217) bound erythrocytes with the same sensitivity to enzyme treatments (Figure 1F).

### Subcellular Localization of Native *Plasmodium falciparum* Surface-Related Antigen in Asexual Stage

**Figure 2.**
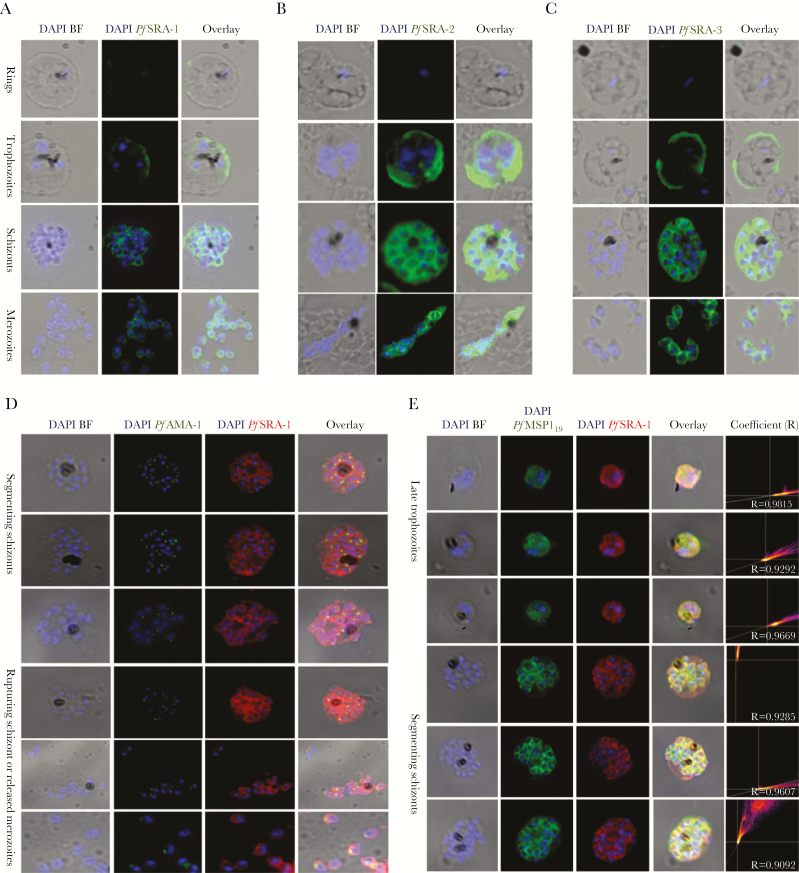
Subcellular localization of native *Plasmodium falciparum* surface-related antigen (*Pf*SRA) in asexual stages. Methanol-fixed 3D7 ring, trophozoite, schizont, and released merozoites were stained with α-*Pf*SRA antibodies. *A*, Anti-*Pf*SRA P1 antibody (green; 1:100). *B*, Anti-*Pf*SRA P2 antibody (green; 1:100). *C*, Anti-*Pf*SRA P3 antibody (green; 1:100). *D*, *Pf*SRA does not colocalize with *Pf*AMA-1 on the merozoite surface. Colabeling of rabbit α-*Pf*SRA-1 (red) with mouse α-*Pf*AMA-1 (1:100; green) antibodies in intact schizonts show that *Pf*SRA is localized on the merozoite surface. Secondary antibodies used are Alexa 488–conjugated goat α-mouse immunoglobulin G (IgG), Alexa 568-conjugated goat α-rabbit IgG (1:200; Life Technologies). Exposure times were identical for all images of the same channel. Nuclei were stained with 4′,6-diamidino-2-phenylindole (DAPI) (blue). *E*, *Pf*SRA colocalizes with *Pf*MSP1_19_ at the parasitophorous vacuolar membrane in late trophozoites and on the merozoite surface. Colabeling of rabbit α-*Pf*SRA-1 (1:100; red) with mouse α-*Pf*MSP1_19_ (1:100; green) antibodies in late trophozoites and intact schizonts show that *Pf*SRA is localized at the PV and on the merozoite surface. Colocalization coefficient (R) is displayed at the far right. Secondary antibodies used are Alexa 488–conjugated goat α-mouse IgG and Alexa 568–conjugated goat α-rabbit IgG (1:200; Life Technologies). Exposure times are identical for all images of the same channel. Nuclei were stained with DAPI (blue). Abbreviations: BP, brightfield; DAPI, 4′,6-diamidino-2-phenylindole; *Pf*SRA, *Plasmodium falciparum* surface-related antigen.

Stage-specific expression analysis in *P. falciparum* asexual stages by IFAs showed that all α-*Pf*SRA peptide antibodies labeled the surface membranes of merozoites in intact schizonts and released merozoites, respectively (Figure 2A–C). However, α-*Pf*SRA antibodies did not label developing ring-stage parasites, which served as a useful internal control for subsequent experiments in asexual stages.

**Figure 3.**
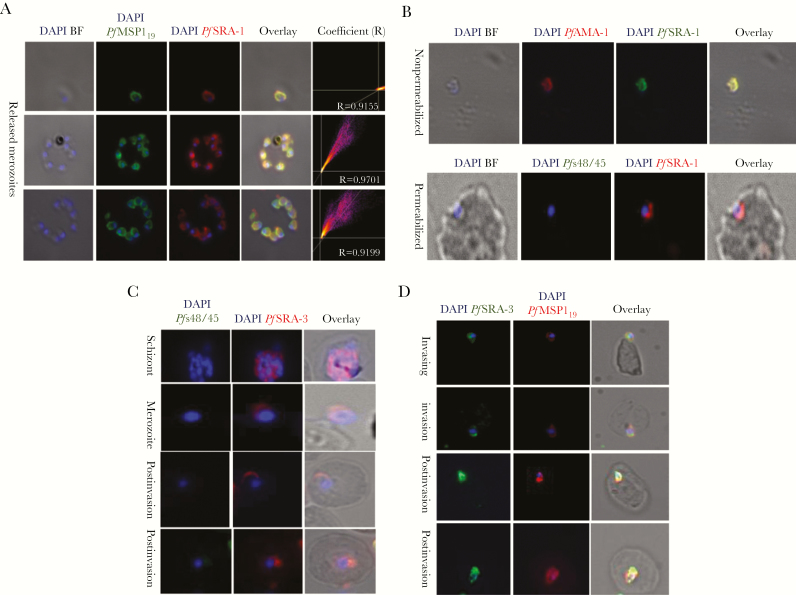
*Plasmodium falciparum* surface-related antigen (*Pf*SRA) localized on the merozoite surface is carried along with the glycosylphosphatidylinositol-anchored MSP1_19_ into invaded erythrocytes. *A*, Colabeling of rabbit α-*Pf*SRA-1 (1:100; red) with mouse α-*Pf*MSP1_19_ (1:100; green) antibodies in released merozoites shows that *Pf*SRA is localized on the merozoite surface. Colocalization coefficient (R) is displayed at the far right. Exposure times are identical for all images of the same channel. Secondary antibodies used are Alexa 488–conjugated goat α-mouse immunoglobulin G (IgG) and Alexa 568–conjugated goat α-rabbit IgG (1:200; Life Technologies). Nuclei were stained with 4′,6-diamidino-2-phenylindole (DAPI) (blue). *B*, Nonpermeabilized, liquid immunofluorescence assays (IFAs) show colabeling of rabbit α-*Pf*SRA-1 (1:100; green) with mouse α-*Pf*AMA-1 (1:100; red) antibodies on the surface of released merozoites. Secondary antibodies used are Alexa 568–conjugated goat α-rat IgG and Alexa 488–conjugated goat α-rabbit IgG (1:200; Life Technologies). Permeabilized IFAs show colabeling of rabbit α-*Pf*SRA-1 (1:100; red) and mouse α-*Pf*s48/45 (green). Secondary antibodies used were Alexa 568–conjugated goat α-rabbit IgG and Alexa 488–conjugated goat α-mouse IgG, (1:200; Life Technologies). Exposure times are identical for all images of the same channel. Nuclei were stained with DAPI (blue). *C*, Colabeling of schizont, released merozoite, and internalized merozoites with rabbit α-*Pf*SRA P3 (red) and mouse α-*Pf*s48/45 (green) show staining of the different parasite stages, but no staining was observed for the gametocyte surface marker, *Pf*s48/45. Nuclei were stained with DAPI (blue). Secondary antibodies used were Alexa 488–conjugated goat α-mouse IgG and Alexa 568–conjugated goat α-rabbit IgG (1:200; Life Technologies). *D*, Invading merozoites were colabeled with α-MSP1_19_ (red) and α-*Pf*SRA P3 (green) to show the internalization of both proteins during invasion, and antibody labeling with both was visible at the development of ring-stage parasites. Secondary antibodies used were Alexa 488–conjugated goat α-mouse IgG and Alexa 568–conjugated goat α-rabbit IgG (1:200; Life Technologies). Abbreviations: BP, brightfield; DAPI, 4′,6-diamidino-2-phenylindole; *Pf*SRA, *Plasmodium falciparum* surface-related antigen.

Colabeling of segmenting or rupturing schizonts and released merozoites with α-*Pf*SRA P1 antibodies and the micronemal marker α-*Pf*AMA-1 showed no colocalization (Figure 2D), even though reports exist about the circumferential staining pattern of *Pf*AMA-1 [27]. However, α-*Pf*SRA P1 antibodies and α-*Pf*MSP1_19_ antibodies colocalized at the parasitophorous vacuole in late trophozoites, segmenting schizonts and released merozoites (Figure 2E). In segmenting schizonts and released merozoites, both proteins colocalized on the merozoite surface (Figures 2E and 3A). Liquid IFA shows that both α-*Pf*SRA P1 antibodies and α-*Pf*AMA-1 antibodies labeled the surface of nonpermeabilized merozoites (Figure 3B). A control panel shows α-*Pf*SRA P1 labeling of an invading merozoite under permeabilized condition, but no labeling was observed for α-*Pf*s48/45 antibodies (Figure 3B).

### Fate and Shedding Patterns of Native *Plasmodium falciparum* Surface-Related Antigen During Erythrocyte Invasion

To determine the fate of native *Pf*SRA during invasion, invading merozoites at different time-points (early, mid, late, and postinvasion) were colabeled with α-*Pf*SRA P3 and α-*Pf*MSP1_19_ antibodies. We observed labeling of both α-*Pf*SRA P3 and α-*Pf*MSP1_19_ antibodies in all of the time points of invasion (Figure 3C and 3D) suggesting that a fragment or the unprocessed forms of *Pf*SRA are carried into erythrocytes during invasion. As a control, late-stage parasites and internalized merozoites were colabeled with α-*Pf*SRA P3 and the gametocyte surface marker α-*Pf*s48/45 antibodies. Whereas α-*Pf*SRA P3 antibody labeled internalized merozoite, no labeling was observed with α-*Pf*s48/45 antibody (Figure 3B).

### Recognition of *Plasmodium falciparum* Surface-Related Antigen Peptides by Human Plasma

**Figure 4.**
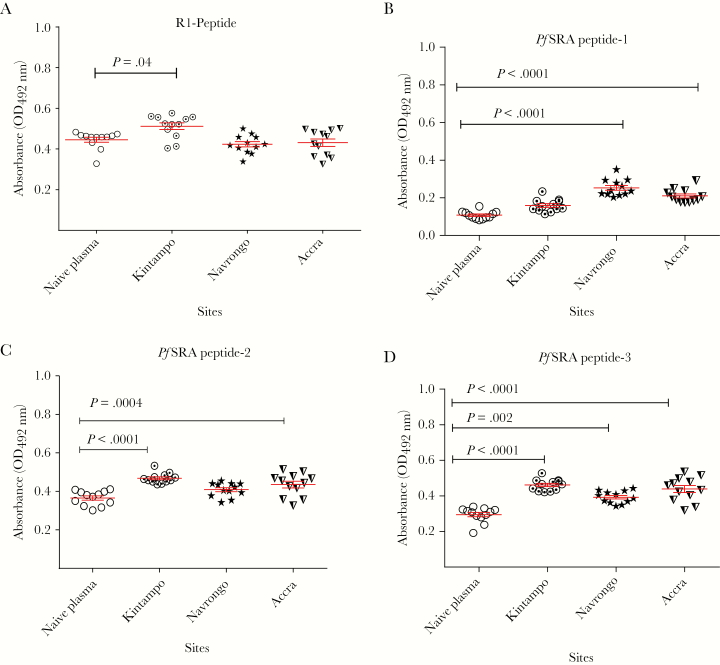
Malaria-exposed children have naturally acquired antibodies against *Plasmodium falciparum* surface-related antigen (*Pf*SRA) synthetic peptides. The reactivity of the 4 synthetic peptides, R1-peptide that blocks *Pf*AMA 1-*Pf*RON4 complex (*A*), *Pf*SRA P1 (*B*), *Pf*SRA P2 (*C*), and *Pf*SRA P3 (*D*) with plasma samples from children with malaria residing in different transmission zones in Ghana (Kintampo, Navrongo, and Accra) were tested by enzyme-linked immunosorbent assay. Naive plasma was used as control. Shown are immunoglobulin G levels in the respective plasma samples at 1:100 dilution, and reactivity was expressed as optical density at 462 nm. Data were analyzed using GraphPad Prism v.6.01 and presented as scatter dot plots summarized as mean and standard error of the mean. Site-to-site comparisons (12 samples per site) were performed using the Krukal–Wallis *H* test with Dunn’s multiple comparison test. The significance of the differences between respective peptide reactivities with plasma samples across the sites was based on the adjusted *P* values (.05). Abbreviations: OD, optical density; *Pf*SRA, *Plasmodium falciparum* surface-related antigen.

A panel of plasma samples from malaria-infected children resident in 3 malaria-endemic sites in Ghana (Accra, Navrongo, and Kintampo) was evaluated for reactivity to *Pf*SRA synthetic peptides by ELISA. Plasma antibodies from children in Accra and Navrongo showed no reactivity to the R1 peptide beyond background (normal human serum [NHS]) levels, whereas those from Kintampo were only slightly above background; however, this difference was statistically significant (*P* = .04) (Figure 4A). Plasma samples from all 3 sites appeared to recognize all 3 *Pf*SRA peptides, and all groups showed reactivity above background levels (Figure 4B–D). Statistically, these differences in reactivity against *Pf*SRA P1 were significant compared with NHS for plasma from Navrongo (*P* < .0001) and Accra (*P* < .0001) but not Kintampo (Figure 4B). Reactivity for *Pf*SRA P2 was significant compared with NHS for plasma from Kintampo (*P* < .0001) and Accra (*P* = .004) but not Navrongo (Figure 4C). However, the reactivity against *Pf*SRA P3 was significant for all 3 sites (Kintampo, *P* < .0001; Navrongo, *P* = .02; and Accra, *P* < .0001) (Figure 4D).

### Recognition of *Plasmodium falciparum* Surface-Related Antigen by Naturally-Acquired Antibodies

**Figure 5.**
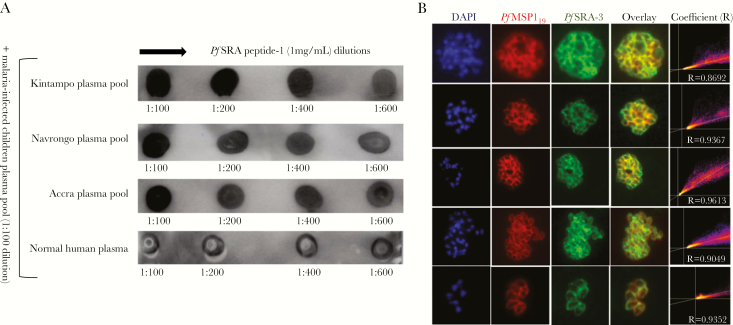
Human *Plasmodium falciparum* surface-related antigen (*Pf*SRA) antibodies recognize the native parasite protein. *A*, Malaria-infected patient plasma from 3 endemic sites (Kintampo, Navrongo, and Accra) in Ghana detected *Pf*SRA peptides by immuno-dot blotting. *B*, Colocalization of α-*Pf*SRA P3 human antibody (green; 1:50) with α-*Pf*MSP1_19_ mouse antibody (red; 1:100) showed that *Pf*SRA is localized on the merozoite surface. Nuclei were stained with 4′,6-diamidino-2-phenylindole (blue). Secondary antibodies used were fluorescein isothiocyanate-conjugated goat α-human immunoglobulin G (IgG; 1:200) and Alexa 568–conjugated goat α-mouse IgG (1:200; Life Technologies). Abbreviations: DAPI, 4′,6-diamidino-2-phenylindole; *Pf*SRA, *Plasmodium falciparum* surface-related antigen.

Consistent with the ELISA data, we also established by immuno-dot blotting that plasma samples from malaria-infected children recognized *Pf*SRA P1 (Figure 5A). Thus, α-*Pf*SRA P3–specific human antibodies against the C-terminus of the protein were purified from a pooled sample of plasma from Kintampo children that showed immunoreactivity (Figure 4D). Consistent with the data from rabbit α-*Pf*SRA antibodies (Figures 2E and 3A), human α-*Pf*SRA P3 immuno-affinity purified antibodies stained schizonts and released merozoites that colocalized with α-*Pf*MSP1_19_ antibodies on the merozoite surface (Figure 5B).

### Subcellular Localization of Native *Plasmodium falciparum* Surface-Related Antigen in Gametocytes

**Figure 6.**
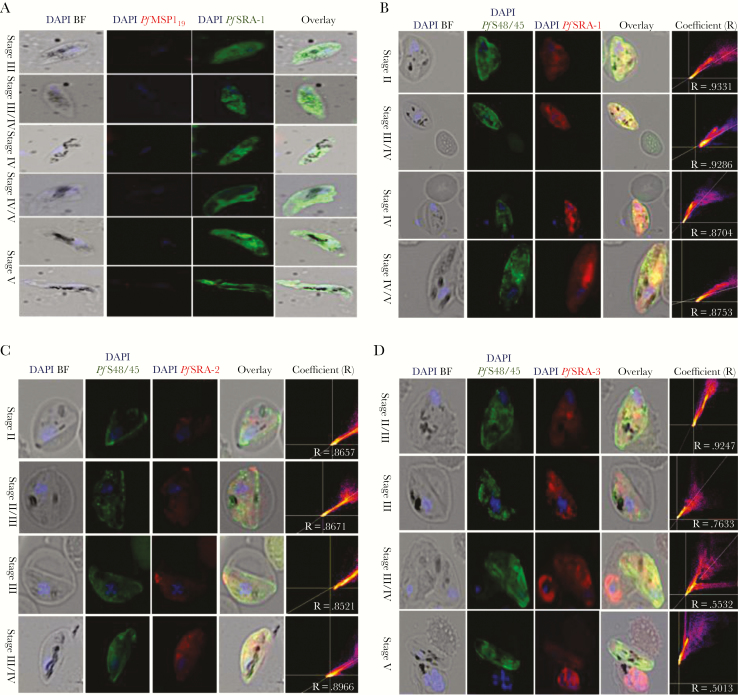
Stage-specific expression of native *Plasmodium falciparum* surface-related antigen (*Pf*SRA) in gametocyte stages. *A*, Methanol-fixed 3D7 gametocytes were stained with anti-*Pf*SRA P3 antibody (red). Nuclei were stained with 4′,6-diamidino-2-phenylindole (DAPI) (blue). *B*, Colabeling of gametocytes (stage II–V) with the respective *Pf*SRA rabbit (α-*Pf*SRA P1; red; 1:100) and mouse (α-*Pf*s48/45; green; 1:100) antibodies. *C*, α-*Pf*SRA P2 (red; 1:100) and mouse α-*Pf*s48/45 (green; 1:100). *D*, α-*Pf*SRA P3 (red; 1:100) and mouse α-*Pf*s48/45 (green; 1:100) suggest gametocyte surface localization for *Pf*SRA. Secondary antibodies used were Alexa 488–conjugated goat α-mouse immunoglobulin G (IgG) and Alexa 568–conjugated goat α-rabbit IgG (1:200; Life Technologies). Nuclei were stained with DAPI (blue). Exposure times in all cases were identical for all images of the same channel. Abbreviations: BP, brightfield; DAPI, 4′,6-diamidino-2-phenylindole; *Pf*SRA, *Plasmodium falciparum* surface-related antigen.

We performed stage-specific expression analysis in gametocytes (stage III–V) by microscopy and observed that α-*Pf*SRA P3 antibodies specifically labeled the membranes of gametocytes, whereas the control antibody (*Pf*MSP1_19_) did not label gametocytes (Figure 6A). Similarly, we colabeled gametocytes (stage II–V) with the respective α-*Pf*SRA peptide antibodies and the gametocyte surface marker α-*Pf*s48/45 antibody and showed close colocalization that appeared to be stage-dependent (Figure 6B–D). Furthermore, *Pf*SRA expression in gametocytes appeared not to be sex-specific, with α-*Pf*SRA P1 antibodies labeling both male and female gametocytes and α-tubulin antibodies labeling only male gametocyte (Supplementary Figure 5).

### Growth Inhibitory Activity of *Plasmodium falciparum* Surface-Related Antigen Peptide-Induced Antibodies

**Figure 7.**
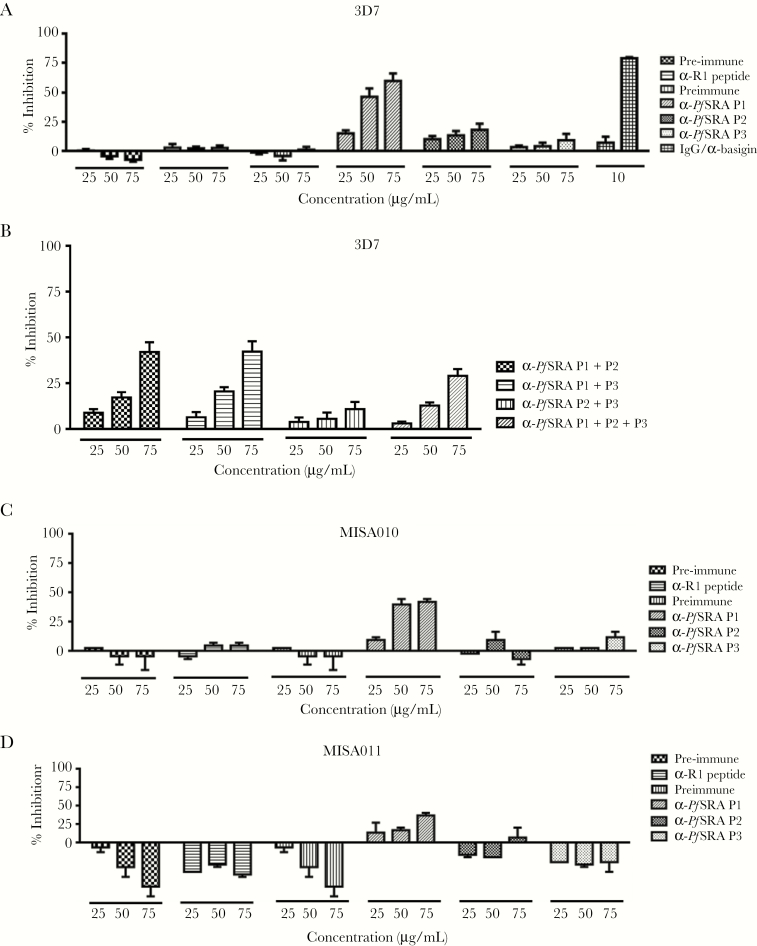
Anti*–Plasmodium falciparum* surface-related antigen (*Pf*SRA) peptide antibodies inhibit erythrocyte invasion of both laboratory strains and clinical isolates. *A*, Anti-*Pf*SRA antibodies were tested at lower concentrations (25–75 μg/mL), and α-*Pf*SRA peptide antibodies exhibited a concentration-dependent inhibition of parasite invasion. Anti-basigin antibodies was used as a positive control, whereas the preimmune sera and R1 peptide antibodies (lot no.: A417040246) were used as negative controls. Data represented are from 2 independent assays performed in duplicate and are shown as means ± SE. *B*, Combinations of α-PfSRA peptide antibodies were tested against *Plasmodium falciparum* 3D7 strain. Preimmune sera and R1 peptide antibodies were used as negative controls. Data represented are from 2 independent assays performed in duplicate and are shown as means ± SE. *C* and *D*, Anti-*Pf*SRA antibodies were tested at lower concentrations (25–75 μg/mL) against *P. falciparum* clinical isolates (MISA010 and MISA 011), and a concentration-dependent inhibition of parasite invasion was observed. Data presented are from 2 independent assays performed in duplicate and are shown as means ± SE. Abbreviation: *Pf*SRA, *Plasmodium falciparum* surface-related antigen.

We evaluated the invasion inhibitory activity of α-*Pf*SRA antibodies against *P. falciparum* 3D7 and W2mef and showed that all *Pf*SRA peptide antibodies exhibited 70%–80% inhibition at 750 μg/mL, whereas the preimmune control did not show any inhibition (Supplementary Figure 6*A* and 6*B*). Because all 3 *Pf*SRA peptide antibodies were inhibitory at higher concentrations (100–750 μg/mL) (Supplementary Figure 6*A* and 6*B*), we have shown that, whereas the preimmune sera and a control immunoglobulin G (IgG) did not inhibit parasite invasion at lower concentrations (25–75 μg/mL), α-*Pf*SRA antibodies exhibited a concentration-dependent inhibition of parasite invasion (Figure 7A). Of all 3 of the *Pf*SRA peptide antibodies tested, only α-*Pf*SRA P1 antibodies showed 60% inhibition of parasite invasion at 75 μg/mL. As a control, anti-Basigin antibodies showed 75% inhibition of parasite invasion at 10 μg/mL (Figure 7A).

To exclude the possibility that serum contaminants might be interfering with parasite invasion, we generated antibodies against a shorter construct of R1-peptide using the same purification procedures for α-*Pf*SRA peptide-specific antibodies, and no inhibition of parasite invasion was observed (Figure 7A). Because all 3 α-*Pf*SRA antibodies were inhibitory at 100–750 μg/mL, we tested combinations of α-*Pf*SRA peptide antibodies against 3D7 and observed that this strategy did not greatly impact on parasite invasion inhibition (Figure 7B). Furthermore, we performed invasion inhibition assays with 2 Ghanaian clinical isolates (MISA010 and MISA011) and observed similar patterns of parasite invasion inhibition for α-*Pf*SRA peptide antibodies (Figure 7C and 7D).

## DISCUSSION

The focus of this work was the identification and functional characterization of a potential blood-stage malaria vaccine candidate (*Pf*SRA). Using Bioinformatics and data-mining analysis of published *P. falciparum* transcriptomes and proteomes [24, 25], we identified *Pf*SRA to be reminiscent of a surface protein, defined by the presence of a signal peptide and a predicted glycosylphosphatidylinositol attachment signal [28, 29]. Indeed, *Pf*SRA has been shown to have *Pf*SUB-1 cleavage sites that were assigned using the Prediction of Protease Specificity analysis platform [25]. The presence of these proteolytic cleavage sites in *Pf*SRA may allude to processing events occurring prior to invasion of erythrocytes. Besides, proteolytic processing in the malaria parasite has been shown to be relevant for cascades of interaction occurring at the parasite–host cell interface [21, 30]. Furthermore, because orthologs are good candidates for multispecies vaccines, we generated sequence alignments with other *Pf*SRA orthologs, which showed that the C-terminus of *Pf*SRA has 5 positionally conserved cysteine residues across the different species orthologs. Our attempt to express the recombinant *Pf*SRA that possesses 67% unstructured regions was unsuccessful. Similarly, others have attempted the expression of the mature recombinant *Pf*SRA in HEK293E cells using a codon-optimized gene (Geneart) and have also been unsuccessful [28]. Considering that *Pf*SRA is an asparagine-rich protein with numerous potential N-linked glycosylation sites, we did not attempt expression in insect cells because the system produces proteins with more complex N-linked glycosylation [31] that may not present the relevant sugar epitopes of native glycosylation [32]. Therefore, the challenges associated with the expression of recombinant *Pf*SRA necessitated the design of chemically synthetized peptides for an epitope-based vaccine strategy.

*Plasmodium falciparum* surface-related antigen harbors coiled-coil domains that are known to be less polymorphic [33, 34], and they form stable structures that elicit functional antibodies that block relevant domains in many organisms [35–37]. Interestingly, these domains have been evaluated as potential targets for peptide-based vaccines [38–40]. The 3 *Pf*SRA peptide antibodies from rabbits specifically detected breakdown products of native *Pf*SRA in ring-stage invasion supernatant or parasite culture supernatant and schizont lysates. This indicated that *Pf*SRA synthetic peptides are antigenic mimics of the native parasite protein. Our data showed that *Pf*SRA is postsynthetically processed by cleavage into parasite culture supernatant, and this is consistent with the *Pf*SUB-1 cleavage sites it harbors. The merozoite surface is remodeled by a series of proteolytic processing events; the physiological relevance of these events in the malaria parasite remains poorly described. However, there are reports suggesting that proteolytic processing may result in activation, structural rearrangement, or acquisition of other new functional properties of native parasite proteins [41].

We have described the fate and shedding pattern of native *Pf*SRA by temporal immunofluorescence imaging using α-*Pf*SRA and α-*Pf*MSP1_19_ antibodies that bound the merozoite surface and were internalized during erythrocyte invasion. Consistent with this observation is a previous report that α-*Pf*MSP1_19_ antibodies were carried into invaded erythrocytes, disrupted intra-erythrocytic development, and inhibited erythrocyte invasion [42–44]. Although the molecular mechanism underlying the internalization of antibodies remains debatable, it was suggested that the tight junction between the merozoite and the erythrocyte might consist of transient interactions that allow the passage of antibodies or surface proteins [19].

Because all rabb3it α-*Pf*SRA peptide antibodies recognized different *Pf*SRA polypeptide fragments in ring-stage invasion supernatant or parasite culture supernatant and schizont lysates at varying thresholds, it was expedient to investigate whether processing could influence differential subcellular localization of *Pf*SRA in the parasite. Interestingly, all 3 rabbit α-*Pf*SRA peptide antibodies showed circumferential association on the merozoite surface at the timing of schizont rupture and merozoite release. Similarly, we performed colabeling in IFAs with gametocytes (stage II–V) using all 3 rabbit α-*Pf*SRA peptide antibodies with the gametocyte surface marker *Pf*s48/45. A clear, punctate rim-fluorescence pattern was observed for all 3 rabbit α-*Pf*SRA peptide antibodies that appeared to colocalize with *Pf*s48/45 in a stage-dependent manner based on the colocalization coefficient. Therefore, the consistency in the staining patterns of all 3 rabbit α-*Pf*SRA peptide antibodies in both asexual and gametocyte stages suggested that proteolytic processing of *Pf*SRA does not cause changes in the subcellular localization of the protein. Although the distribution of proteins in male or female gametocytes could be linked to functional divergence between the sexes [45], we observed the expression of *Pf*SRA in both male and female gametocytes. Consistent with this observation is an existing report on *Plasmodium berghei* gametocyte egress and sporozoite traversal protein (*Pb*GEST) expression in both sexes [46].

Also, it was imperative to determine whether *Pf*SRA in released merozoites was accessible to humoral immune surveillance during the short period of erythrocyte invasion. Our serological screens, buttressed by immuno-dot blot assays, with plasma samples from malaria-infected children residing at different endemic sites showed differences in total IgG recognition frequencies for *Pf*SRA peptides. This could be linked to varying transmission intensity rates as reported for samples collected from different endemic sites in previous studies [33, 47]. In most cases, the reactivity of all 3 *Pf*SRA synthetic peptides was low, and the likely explanation for this could be the hindered accessibility of *Pf*SRA in the native context. However, our data from IFAs showed that the immuno-affinity purified, human α-*Pf*SRA peptide antibodies labeled native *Pf*SRA, which suggests that malaria-infected populations have naturally acquired antibodies against *Pf*SRA.

Generally, several receptors have been characterized based on their sensitivities to different enzyme treatments. Notably, neuraminidase removes sialic acids from glycophorins, trypsin cleaves peptide backbones of several receptors (glycophorin A, glycophorin C, and complement receptor 1), and chymotrypsin cleaves glycophorin B and complement receptor 1, among others [48]. We have shown that the 32-kDa–processed fragment of native *Pf*SRA binds normal human erythrocytes, but the molecular identity of the receptor remains unknown. However, we have classified the putative receptor for *Pf*SRA as sialic acid–dependent on the basis of its binding specificity, which is sensitive to treatments with both neuraminidase and trypsin but resistant to chymotrypsin, a binding phenotype that fits the description of the receptor glycophorin C. The enzyme sensitivity profile of *Pf*SRA binding to erythrocytes is similar to that observed for *Pf*EBA-140 (region II), the parasite ligand for glycophorin C [49]. Additional investigations are required to determine whether *Pf*SRA also interacts with glycophorin C, possibly via a different binding site.

Our data revealed that *Pf*SRA peptides induce functional antibodies that inhibited *P. falciparum* erythrocyte invasion of both laboratory strains and clinical isolates. The observed invasion inhibitory activity of rabbit α-*Pf*SRA peptide antibodies could be attributed to indirect effects of antibody binding to the merozoite surface or direct inhibition of proteolytic processing events. The demonstration that *Pf*SRA synthetic peptides induced erythrocyte invasion inhibitory antibodies and the successful purification of a limited amount of *Pf*SRA-specific human antibodies from patient plasma suggested that the synthetic peptides possessed structural integrity or conformation that mimics the native *Pf*SRA.

In summary, this study has provided relevant new information regarding the proteolytic processing of *Pf*SRA that supports the idea of targeting these cleavage events for development of antimalarial therapies. The expression of *Pf*SRA in late stages of gametocytes is an unprecedented opportunity that should be explored for potential transmission-blocking vaccines. Also, *Pf*SRA-specific immune responses triggered in natural infections may inform the inclusion of *Pf*SRA as a candidate for epitope-based, blood-stage malaria vaccine development.

## Supplementary Data

Supplementary materials are available at *The Journal of Infectious Diseases* online. Consisting of data provided by the authors to benefit the reader, the posted materials are not copyedited and are the sole responsibility of the authors, so questions or comments should be addressed to the corresponding author.

Supplementary Figures

## Notes

***Acknowledgements.*** We thank Dr Anthony Holder for providing MSP1_19_ antibody and the West African Centre for Cell Biology of Infectious Pathogens (WACCBIP) for providing anonymized archived plasma sample from children resident at Kintampo, Navrongo, and Accra. Anti-*Plasmodium falciparum* 48/45-kDa Gamete Surface Protein (Pfs48/45) monoclonal antibody was contributed by Louis H. Miller and Alan Saul through the National Institute for Allergy and Infectious Diseases (NIAID) BEI Resources (product number MRA-316A). Erythrocyte Binding Antigen, Region II was also contributed by NIAID BEI Resources (product number MRA-1162).

***Disclaimer.***  The views expressed in this publication are those of the author(s) and not necessarily those of the African Academy of Sciences, the New Partnership for Africa’s Development Planning and Coordinating Agency, the Wellcome Trust, or the UK government.

***Financial support.*** This work was supported by funds from a World Bank African Centres of Excellence grant (ACE02-WACCBIP to G. A. A.) and a Developing Excellence in Leadership Training and Science (DELTAS) Africa grant (DEL-15-007 to G. A. A.). E. A. and Y. A. are WACCBIP-Wellcome Trust DELTAS Postdoctoral Fellows. The DELTAS Africa Initiative is an independent funding scheme of the African Academy of Sciences’s Alliance for Accelerating Excellence in Science in Africa and supported by the New Partnership for Africa’s Development Planning and Coordinating Agency with funding from the Wellcome Trust (107755/Z/15/Z to G. A. A.) and the UK government.

***Potential conflicts of interest.*** All authors: No reported conflicts of interest. All authors have submitted the ICMJE Form for Disclosure of Potential Conflicts of Interest. Conflicts that the editors consider relevant to the content of the manuscript have been disclosed.
