# Supplementary material for: Functional Characterization of Plasmodium falciparum Surface-Related Antigen as a Potential Blood-Stage Vaccine Target
Source: J Infect Dis. 2018 Apr 18;218(5):778–90. doi: 10.1093/infdis/jiy222 (PMC6057521; doi:10.1093/infdis/jiy222)
Supplement: Supplementary Figures [file jiy222_suppl_supplementary_figures.docx]

**Supplementary Figures**

**Figure S1**

**Figure S1: Systematic screens for uncharacterized *P. falciparum* invasion-related proteins evealed *Pf*SRA as one of the top hits that emerged.**

**Figure S2**

**
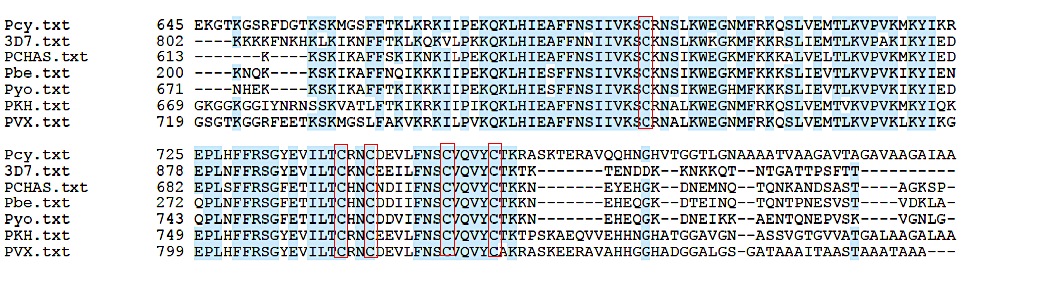
**

**Figure S2: Sequence conservation of *Pf*SRA.**

**Figure S3**


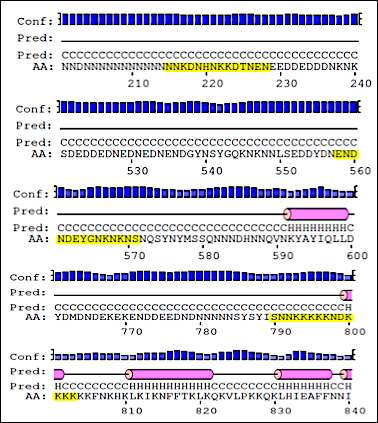


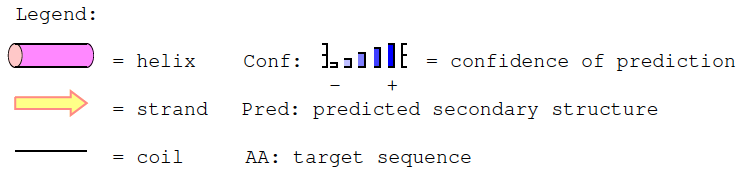


**Figure S3: *Pf*SRA secondary structure prediction.**

**Figure S4**

**Figure S4: Detection of the erythrocyte binding function of the full-length *Pf*SRA**.

**Figure S5**

**
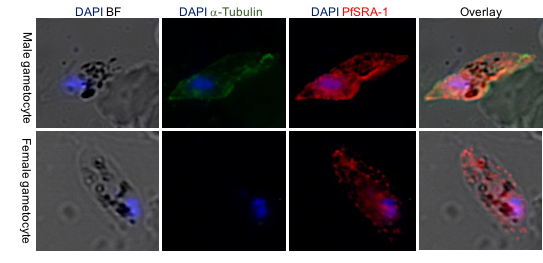
**

**Figure S5: *Pf*SRA labels both male and female gametocytes**.

**Figure S6**

**Figure S6: *Pf*SRA antibodies exhibit parasite growth inhibitory activity**.
